# Tezepelumab After Mepolizumab in Allergic Bronchopulmonary Mycosis: A Case Report

**DOI:** 10.7759/cureus.98359

**Published:** 2025-12-03

**Authors:** Naohiro Oda, Tomohiko Oka, Ryoko Tsuji, Tetsuya Takeguchi, Atsushi Shimonishi, Akihiko Taniguchi, Reo Mitani, Ichiro Takata

**Affiliations:** 1 Department of Internal Medicine, Fukuyama City Hospital, Fukuyama, JPN

**Keywords:** airway mucus plugs, allergic bronchopulmonary aspergillosis, allergic bronchopulmonary mycosis, tezepelumab, thymic stromal lymphopoietin

## Abstract

We describe the case of a 43-year-old woman with allergic bronchopulmonary mycosis (ABPM) and severe asthma who improved clinically on mepolizumab with eosinophil suppression but had recurrent mucus plugs, persistent right-middle-lobe atelectasis, rising fractional exhaled nitric oxide (FeNO), and high total IgE levels. Following a switch to tezepelumab, she attained sustained symptom control, no systemic steroid-requiring exacerbations, substantial FeNO and IgE decline, and radiologic reduction of mucus plugs and atelectasis. This case illustrates that residual upstream type 2 activity can persist despite IL-5 blockade, and thymic stromal lymphopoietin (TSLP) inhibition may address this gap by concurrently attenuating multiple effectors. In mucus-predominant allergic bronchopulmonary aspergillosis/ABPM with high FeNO/IgE levels after anti-IL-5 therapy, phenotype- and biomarker-guided sequencing of anti-TSLP antibodies may be considered.

## Introduction

Allergic bronchopulmonary aspergillosis (ABPA) and the broader entity allergic bronchopulmonary mycosis (ABPM) are characterized by severe asthma, recurrent pulmonary infiltrates, central bronchiectasis, and eosinophilic mucus plugs. Contemporary frameworks, including the 2021 Japanese ABPM/ABPA criteria and the 2024 revised International Society for Human and Animal Mycology (ISHAM) guidelines, have standardized diagnostic approaches and recognized distinct radiologic phenotypes, such as “ABPA with mucus plugging,” which is associated with increased disease activity [1,2].

For acute disease, oral glucocorticoids or itraconazole are recommended, with combination therapy considered in cases of relapse [2]. In parallel, biologic agents that target the type 2 (T2) inflammatory pathway, such as anti-IgE, anti-IL-5/IL-5Rα, anti-IL-4Rα, and anti-thymic stromal lymphopoietin (TSLP), are increasingly used to reduce systemic corticosteroid exposure. Among these, the most extensive experience exists for omalizumab, while growing real-world and case-based evidence supports the use of other agents [2-9]. Fungal allergens stimulate the airway epithelium to release alarmins, such as TSLP, which condition dendritic cells and activate type 2 innate lymphoid cells, thereby sustaining upstream T2 signaling [10,11]. Consistent with this biology, anti-IL-5/IL-5Rα therapies reduce exacerbations and decrease mucus plugs in many patients with ABPA; however, total IgE levels often remain unchanged, suggesting residual upstream activity in mucus-predominant phenotypes [4]. Clinical improvement with anti-IL-4Rα after anti-IL-5/IL-5Rα exposure has been reported [5,6], and emerging evidence also supports the efficacy of TSLP inhibition, including in patients previously treated with anti-IL-5 agents [7-9].

Here, we describe a patient with ABPM and radiologic evidence of mucus plugs whose disease control was incomplete under anti-IL-5 therapy but improved after switching to the anti-TSLP antibody tezepelumab, with concordant declines in fractional exhaled nitric oxide (FeNO), total IgE levels, and mucus plugs.

## Case presentation

**Figure 1:**
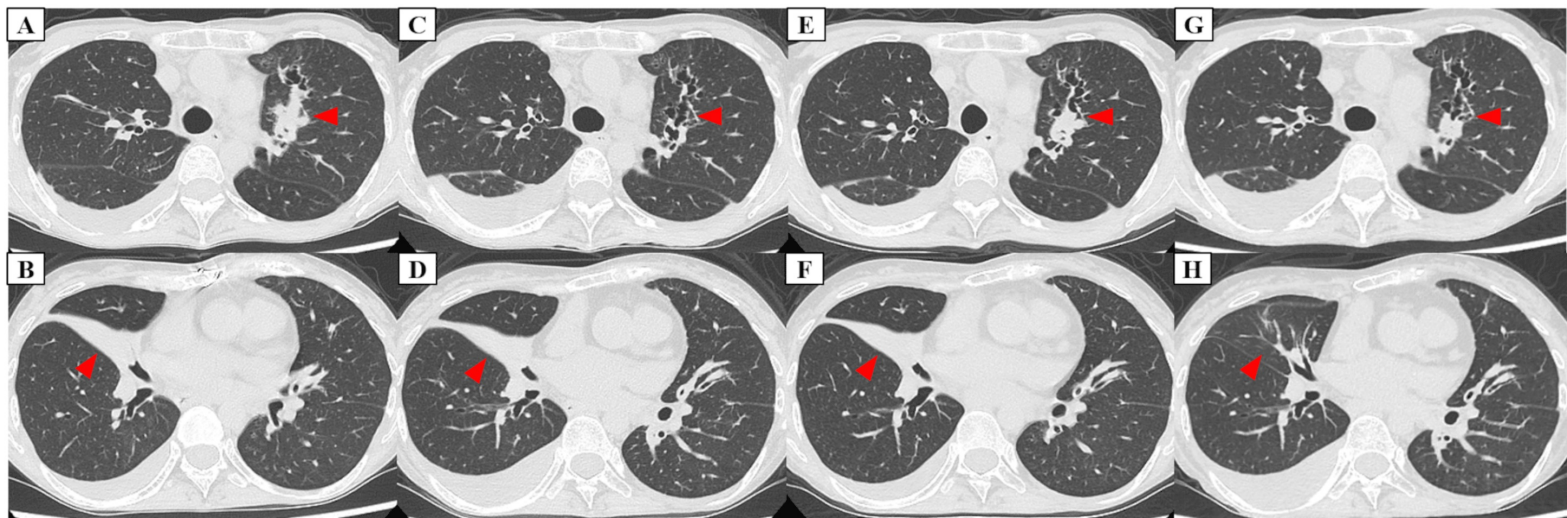
Serial chest CT changes during mepolizumab and after switch to tezepelumab. At the start of mepolizumab therapy (A, B), chest CT shows central bronchiectasis with segmental mucus plugs in dilated bronchi of the left upper lobe (LUL) and consolidation with atelectasis in the right middle lobe (RML). Twelve months after starting mepolizumab (C, D), the LUL mucus plug burden had decreased. Fifteen months after starting mepolizumab (E, F), LUL mucus plugs recurred. Six months after starting tezepelumab (G, H), the LUL mucus plug burden had further decreased, and the prior RML atelectasis/consolidation had resolved.

A 43-year-old Japanese woman with childhood-onset asthma, allergic rhinitis, atopic dermatitis, and cholinergic urticaria (never smoker) presented with uncontrolled severe asthma despite receiving high-dose budesonide/formoterol and tiotropium. Her medical history included hysterectomy with bilateral oophorectomy for bilateral ovarian tumors and multiple uterine fibroids, as well as prior cardiac surgery for pulmonary artery stenosis. At baseline, the Asthma Control Test (ACT) score was 13, the blood eosinophil count was elevated, the total IgE level was markedly increased, and the FeNO level was high (Table 1). Specific IgE tests were positive for *Aspergillus fumigatus*, *Penicillium*, and *Candida*. Because adequate sputum samples could not be obtained despite repeated attempts, neither fungal culture nor antifungal susceptibility testing was performed. Both myeloperoxidase-specific antineutrophil cytoplasmic antibody and proteinase 3 antineutrophil cytoplasmic antibody tests were negative. Chest CT revealed central bronchiectasis with segmental mucus plugging in the dilated bronchi of the left upper lobe and consolidation with atelectasis in the right middle lobe (RML) (Figures 1A, 1B). The patient met both the Japanese ABPA/ABPM criteria and the revised ISHAM-ABPA criteria [1,2].

**Table 1:** Longitudinal symptom scores, biomarkers, and lung function across mepolizumab and subsequent tezepelumab therapy. Reference ranges/interpretation: ACT scores range from 5 to 25 points, with scores ≥20 indicating well-controlled asthma. Total IgE, 0–170 IU/mL (reference interval in our laboratory). Mepo: mepolizumab; Teze: tezepelumab; mo: months; ACT: Asthma Control Test; FeNO: fractional exhaled nitric oxide; FEV1: forced expiratory volume in one second; MMEF: maximal mid-expiratory flow; Eos: blood eosinophil count; —: not obtained at that visit

	Mepo initiation (month 0)	Mepo +3 mo	Mepo +6 mo	Mepo +9 mo	Mepo +12 mo	Mepo +15 mo (Teze initiation)	Teze +3 mo	Teze +6 mo	Teze +9 mo
ACT	13	18	20	23	23	20	19	24	22
FeNO (ppb)	292	282	174	114	162	152	63	64	41
FEV1 (L)	2.10	1.95	2.10	—	2.04	—	2.06	—	2.02
%FEV1 (%)	87.5	81.3	87.5	—	86.8	—	86.6	—	85.6
MMEF (L/second)	1.84	1.95	2.15	—	2.32	—	2.25	—	2.3
%MMEF (%)	57.0	60.4	66.6	—	73.2	—	70.3	—	72.8
Total IgE (IU/mL)	1,484	1,701	1,703	—	1,917	1,417	1,910	1,062	1,132
Eos (/µL)	526	48	35	56	38	29	74	40	49

Given the history of long-term intermittent systemic corticosteroid use for asthma and premature menopause following hysterectomy with bilateral oophorectomy, resulting in severe osteoporosis, ABPM-directed systemic corticosteroids were avoided. Instead, biologic therapy was initiated for uncontrolled severe asthma.

Mepolizumab (100 mg every four weeks) was started. After initiation, symptoms improved, the ACT score increased, no exacerbations requiring systemic corticosteroids occurred, and peripheral eosinophils were suppressed. The maximal mid-expiratory flow, a small airway flow index, increased from a baseline of 57% predicted. However, after an initial decline, FeNO increased again. Chest CT demonstrated re-accumulation of mucus in the left upper lobe, and RML atelectasis showed no clear improvement during mepolizumab therapy (Figures 1C-1F). Total IgE levels remained high (Table 1).

As residual upstream type 2 inflammation was suspected, treatment was switched to tezepelumab (210 mg every four weeks). Subsequently, no exacerbations requiring systemic corticosteroids occurred, ACT entered and remained within the well-controlled range, FeNO declined substantially, and total IgE levels decreased (Table 1). Follow-up chest CT showed a reduction of mucus plugs in the left upper lobe and marked improvement of the prior RML atelectasis and consolidation (Figures 1G, 1H).

Throughout the entire observation period, from the initiation of mepolizumab to the final follow-up visit during treatment with tezepelumab, inhaled maintenance therapy remained unchanged with good adherence. During this time, neither antifungal agents nor systemic corticosteroids were administered.

## Discussion

This case illustrates that downstream eosinophil suppression under IL-5 blockade can coexist with ongoing upstream T2 activity, as evidenced by persistent FeNO/IgE elevation and the presence of mucus plugs. We show that targeting the epithelial alarmin TSLP may help bridge this therapeutic gap. TSLP is released from the airway epithelium in response to allergens, viral infection, and type 2 cytokines, and it activates dendritic cells and ILC2s to promote Th2 inflammation, positioning it upstream of the IgE, IL-5, and IL-13 pathways [10,11]. The pattern observed here parallels real-world ABPA data, where anti-IL-5/IL-5Rα therapies reduce exacerbations and diminish plugs but often fail to lower IgE levels [4]. In ABPA, consistent with allergic airway biology, persistent epithelial- and alarmin-derived signaling, together with IL-13-associated goblet cell metaplasia, MUC5AC-rich secretions, and eosinophil extracellular trap formation, likely contribute to tenacious mucus despite suppression of peripheral eosinophils [12,13].

At the time initial therapy was selected, more clinical experience and published evidence were available for anti-IL-5/IL-5Rα agents in ABPA/ABPM, and the patient’s prominent eosinophilic phenotype supported IL-5 blockade as a reasonable first-line choice. Tezepelumab inhibits TSLP and consequently attenuates multiple downstream effectors, including IgE, IL-5, and IL-13 levels. In randomized trials, tezepelumab reduced blood eosinophils, FeNO, and total IgE levels concurrently, consistent with upstream modulation [10,14]. Notably, randomized CT analysis demonstrated a significant reduction in occlusive airway mucus plugs with tezepelumab, correlating with improved lung function and eosinophil-related biomarkers, which align mechanistically with the radiologic clearance observed in this patient [15].

Although both dupilumab and omalizumab would also have been a reasonable option, several factors supported the selection of tezepelumab: a plug-dominant disease with persistently high FeNO/IgE levels despite IL-5 blockade; trial-level evidence that anti-TSLP concurrently lowers eosinophils, FeNO, and IgE levels while reducing mucus plugs [14,15]; concerns regarding dupilumab-associated transient hypereosinophilia in asthma and rare eosinophilic pneumonia in ABPA [16,17]; and practical constraints on omalizumab dosing in patients with very high IgE levels [3].

Our patient’s subsequent course, including the decline in FeNO, reduction in IgE levels, and radiologic improvement after the switch, is consistent with upstream pathway modulation and complements emerging evidence. Although natural variability cannot be excluded, inhaled maintenance therapy remained unchanged with good adherence, and neither antifungal agents nor systemic corticosteroids were administered throughout the observation period, supporting a treatment effect temporally associated with tezepelumab initiation.

In ABPA/ABPM, antifungal agents are an important therapeutic option, particularly in patients with acute disease, relapsing or steroid-dependent disease, or clear evidence of fungal infection. However, evidence to support their routine use in all patients is limited, and current guidelines place oral itraconazole as an option for acute or relapsing disease, especially when systemic corticosteroids are required or difficult to taper [2]. In our patient, itraconazole monotherapy could have been considered as a steroid-sparing strategy, but we prioritized biologic therapy because she had uncontrolled severe asthma with a pronounced type 2 phenotype. Systemic corticosteroids were intentionally avoided owing to severe osteoporosis, prior long-term steroid exposure, and premature menopause, and disease activity remained controlled with biologic therapy alone. As there were no clinical or radiologic features suggesting acute ABPA/ABPM exacerbation or invasive fungal disease, we judged that add-on antifungal therapy was not mandatory in this setting and decided not to introduce antifungal treatment.

Ogata et al. reported the case of an 82-year-old man with ABPA who developed a high-attenuation mucus plug and lobar collapse while on mepolizumab; switching to tezepelumab yielded radiologic remission within three months and a reduction in IgE, although FeNO and spirometry were not available [7]. Matsuno described a single successful tezepelumab case [8], and Yamaguchi et al. reported three biologic-naïve ABPA patients showing consistent improvements in symptoms, oral corticosteroid tapering, and resolution of mucus plugs on imaging [9]. Compared with previous reports, our case describes a younger female with multifungal sensitization who received sequential anti-IL-5 and anti-TSLP therapy, with concordant reductions in FeNO and total IgE levels and steroid-free disease control.

Together, these observations support a phenotype- and biomarker-guided sequencing approach in ABPA/ABPM. In patients with mucus plugs and elevated FeNO/IgE levels despite IL-5 blockade, upstream inhibition may be considered before further intensifying downstream agents. Nonetheless, as current evidence for tezepelumab in ABPA/ABPM is limited to case reports and small series, larger cohorts and prospective studies are needed to define optimal patient selection and comparative positioning relative to other biologic therapies.

## Conclusions

In ABPA/ABPM, the steroid-sparing potential of biologics is clinically significant, as systemic glucocorticoids are often undesirable. When a partial response to anti-IL-5 coexists with mucus plugs and elevated FeNO/IgE levels, switching to TSLP inhibition may yield additional biomarker improvements and radiological benefits without the need for systemic corticosteroids. This case adds to the existing literature and supports the use of phenotype- and biomarker-guided sequencing strategies in difficult cases of ABPA/ABPM.
